# The Influence of Syringic Acid and Erucic Acid on the Antioxidant Properties of Natural Rubber: Experimental and Molecular Simulation Investigations

**DOI:** 10.3390/polym14204254

**Published:** 2022-10-11

**Authors:** Shihao Chen, Xiujuan Wang, Xueting Wang, Wei Zheng, Shaojian He, Meng Song, Hongzhen Wang

**Affiliations:** 1 Key Laboratory of Rubber-Plastics, Ministry of Education/Shandong Provincial Key Laboratory of Rubber-Plastics, Qingdao University of Science & Technology, Qingdao 266042, China; 2 School of International Education, Beijing University of Chemical Technology, Beijing 102202, China; 3 Beijing Key Laboratory of Energy Safety and Clean Utilization, North China Electric Power University, Beijing 102206, China; 4 School of Materials and Chemical Engineering, Zhongyuan University of Technology, Zhengzhou 450007, China

**Keywords:** natural phenolic antioxidants, natural rubber, quantum mechanics simulation, molecular dynamics simulation, antioxidative activity

## Abstract

In this work, the influence of syringic acid (SA) and erucic acid (EA) on the oxidation resistance of natural rubber (NR) was investigated by combining experimental and computational methods. The antioxidant activities of SA and EA were predicted by calculating the enthalpy of bond dissociation (*BDE*), the anti-migration ability of antioxidants (AOs) in the rubber matrix by calculating the mean square displacement (*MSD*), and the effect of antioxidants on oxygen barrier properties of rubber materials by calculating the permeability coefficient (*P*). The predicted result is that EA has a better comprehensive performance than SA. The DPPH (2,2-diphenyl-1-picrylhydrazyl) test and mechanical properties test demonstrated the results predicted by the simulations. Both SA and EA can protect natural rubber, while EA has a better comprehensive effect.

## 1. Introduction

Natural rubber (NR), with excellent resilience, insulation, and plasticity, is indispensable in aerospace [1], military [2], medical [3,4], and other industrial fields [5,6]. NR applications include gloves, sports equipment, tires, shoe soles, etc. [7,8]. However, the largest limitation of NR is the aging problem. The existence of numerous side methyl groups, active hydrogens, and unsaturated carbon chains render NR products aging easily, especially in the environment of light, heat, and oxygen [9,10,11]. Thermo-oxidative aging, the most prevalent of the many types of aging, may lead to the degradation of the physical properties of NR, thus limiting the practical application of NR products and shortening its service life [12]. Therefore, it is significant to improve the anti-aging performance of NR products to prolong their service life and expand their application scope.

In general, antioxidants, as effective additives, are the most effective and convenient way to protect rubber materials from aging [13]. However, many industrial antioxidants such as the phenolic antioxidant butylated hydroxyanisole (BHA) [14] or butylated hydroxytoluene (BHT) [15] and the amine antioxidants *N*-cyclohexyl-*N*′-phenylbenzene-1,4-diamine (4010NA) [16] or *N*-(1,3-Dimethylbutyl)-*N*-Phenyl-1,4-Benzenediamine (4020) [17], are unsafe, which failed to obtain EU REACH environmental protection approval. Synthetic antioxidants can effectively protect rubber products, but toxicity and environmental pollution still need to be solved [18].

Nowadays, for reasons of environmental protection, researchers prefer to study natural antioxidants [19]. A variety of natural compounds can be used as antioxidants, for example, ascorbic acid, phenolic acid, flavonoids, tocopherol, etc. [20]. It is a difficult and cumbersome problem for researchers to find suitable antioxidants for rubber materials with different needs [21]. Most research studies have demonstrated that the hydrogen (-H) bond dissociation energy (*BDE*), molecular structure, and some physical factors are important in influencing the antioxidant effect of AO [22]. Specifically, these physical factors are associated with the migration and dispersion of antioxidants (AOs) [23]. However, most studies on the antioxidant effect of AOs are qualitatively investigated through traditional aging experiments. The quantitative study of the antioxidant properties of natural AOs at the microscopic molecular level is still inadequate [24].

Molecular simulation is an economical and effective scientific research tool that can quantitatively analyze the microstructure and performance of molecules [25]. For instance, the free energy of dissociation can be computed from the chemical reaction perspective by quantum mechanical (QM) methods [26]. The molecular dynamics (MD) method has been used to calculate some physical parameters, such as solubility parameter (*δ*), solution coefficient (*S*), diffusion coefficient (*D*), and mean square displacement (*MSD*) [27]. Yu et al. [28] The *BDE* of three different vitamins was quantitatively calculated by QM simulations based on the DFT method to predict their anti-oxidation activity so as to explore the potential mechanism of antioxidants. In the work of Haidasz et al. [29], the dissociation energy of two kinds of triphenol antioxidants and the reaction energy barrier between antioxidants and alkyl peroxy radicals were calculated by QM simulation. Therefore, molecular simulation can be used to calculate some relevant parameters of antioxidants from the molecular level [30].

In this work, we aimed to study the influence of SA and EA on the antioxidant properties of natural rubber. Through the combination of computational simulation and experiment, we will study the factors affecting the antioxidant activity of antioxidants from the following aspects (i.e., free energy of antioxidant dissociation reaction, compatibility and migration resistance of antioxidants in NR, and the barrier properties of O_2_).

## 2. Molecular Simulations

### 2.1. Quantum Mechanics (QM) Simulation

**Figure 1:**
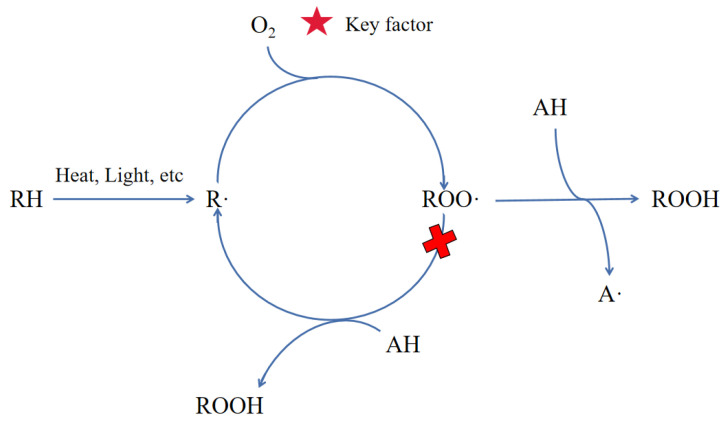
Thermo-oxidative aging process of RH and the anti-oxidation reaction of AH.

Figure 1 reveals the autoxidation process of the rubber chain (RH) and the mechanism of phenolic antioxidants to prevent further oxidation of RH. Antioxidants (AHs) can provide hydrogen radicals (H•) to react with the peroxyl radical (ROO•) and prevent ROO• from further oxidizing RH [31]. The chain reaction is terminated; thus, the antioxidant functioned as an inhibitor of RH oxidation [32]. Therefore, the ability of antioxidants to provide active hydrogen atoms (H•) reflects the antioxidant activity of antioxidants. Generally, the lower the *BDE* value of the antioxidant, the more likely it is to contribute hydrogen radical to ROO•, so *BDE* is a very important parameter for evaluating the antioxidant activity of antioxidants [33].

All QM simulations are performed on the Dmol^3^ module of Materials Studio (MS) software based on density general function theory (DFT). The Kohn–Sham (K-S) equation is proposed to solve the problem of DFT in practical applications. The calculation of the exchange-correlation energy in the K-S equation utilizes the Perdew–Burke–Ernzerhof (PBE) general function within the generalized gradient approximation (GGA). The wave function of the system is described by the Triple Numerical plus polarization (TNP) basis set [33]. To obtain a fine convergence quality criterion, the self-consistent field (SCF) procedure was applied, and its convergence threshold of 10^−6^ au. In the calculation of radical energy, the spin multiplicity of the system should be set to “Doublet” because of the presence of a single electron.

**Figure 2:**
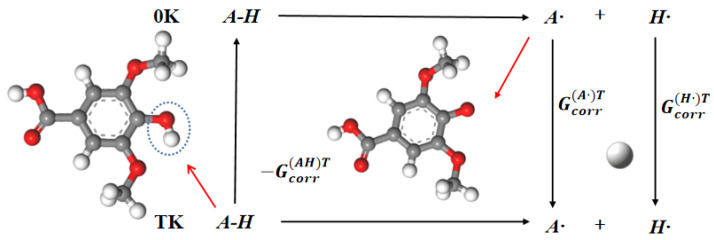
Example of the thermodynamic cycle for SA. (White spheres, red spheres, and gray spheres represent H atoms, O atoms, and C atoms, respectively).

All structures, including antioxidants (AHs), antioxidants free radicals (A·), and hydrogen atoms (H·), as shown in Figure 2, were optimized with convergence tolerance of 10^−5^ au for energy, 0.002 Hartree/Å for maximum force, and 0.005 Å for maximum displacement. The calculated energy is electronic energy at 0 K [27]. The energy at other temperatures (*T*) is determined with the help of the thermodynamic cycle, and Figure 2 shows the cycle with the example of SA. Thus, the *BDE* value can be obtained from the following equation [34].

(1) $$\Delta G^{T}=\left[E\left(A\cdot \right)+G_{corr}^{\left(A\cdot \right)T}\right]+\left[E\left(H\cdot \right)+G_{corr}^{\left(H\cdot \right)T}\right]-\left[E\left(AH\right)+G_{corr}^{\left(AH\right)T}\right]\;$$

where Δ*G^T^* is the *BDE* value at a certain temperature *T*. *E*(*A*·) represents the energy of *A*· at 0 K, *E*(*H*·) represents the energy of H· at 0 K, and *E*(*AH*) represents the energy of *AH* at 0 K. $G_{corr}^{\left(A\cdot \right)T}$, $G_{corr}^{\left(H\cdot \right)T},$ and $G_{corr}^{\left(AH\right)T}$ are energy corrections at specific *T* K.

### 2.2. Molecular Dynamics (MD) Simulation

**Figure 3:**
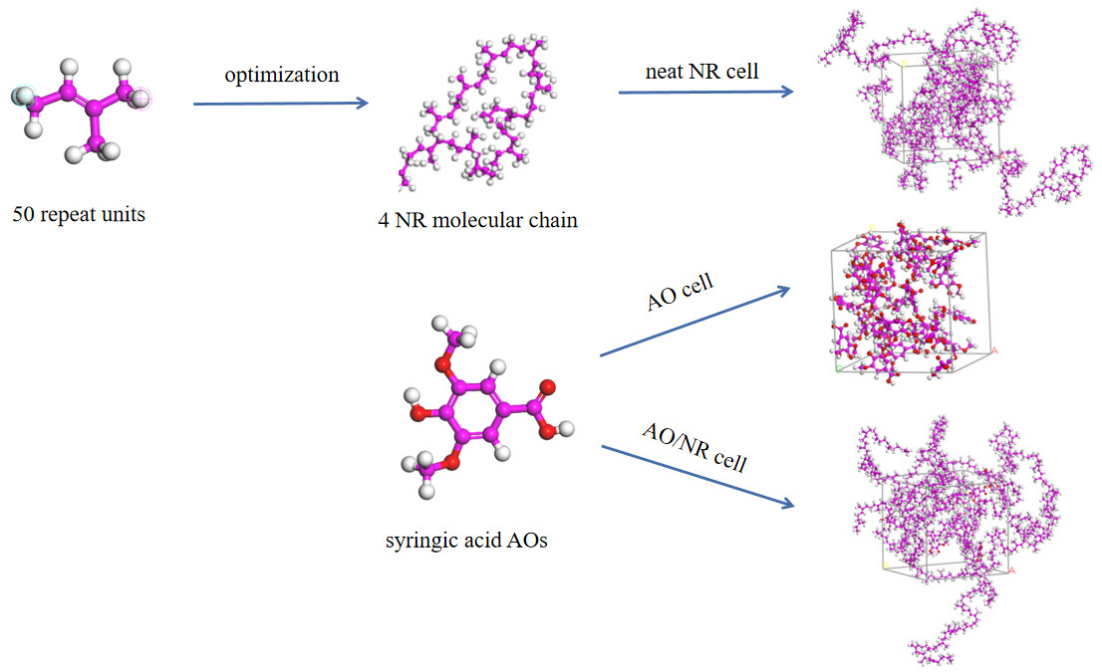
Construction processes of three types of amorphous cells (take syringic acid, for example, and the purple, white, and red spheres represent C atoms, H atoms, and O atoms, respectively).

Solubility parameters (*δ*) of AOs and NR were calculated in MD simulations to analyze the compatibility of antioxidants. The migration ability of AOs in rubber products was investigated by calculating the mean square displacement (*MSD*). The value of *MSD* indicates the migration ability of antioxidants in the NR matrix, and the smaller the value of *MSD*, the more resistant the antioxidants are to migration. The permeability coefficient (*P*) was used to analyze the process of O_2_ entry into the rubber materials. The whole MD simulation was performed under the amorphous cell, and the COMPASSII force field was used [35]. Figure 3 shows the construction process of the three cell models. In the NR and AO system, for example, 4 NR molecular chains containing 50 repeat units and 5 antioxidant molecules were placed into a cell. Then, the cells were first relaxed by geometric optimization using intelligent methods to obtain low potential energy [27]. A range of procedures was carried out in order to achieve a complete equilibrium for all cells. In addition, the cell experienced 100 annealing cycles through a combination of NVE ensemble (constant number of atoms, constant volume, and constant energy) with annealing temperatures from 298 to 500 K. After that, the annealed cell was further relaxed by passing through an NVT ensemble at a constant temperature of 200 ps, and an NPT ensemble at a constant pressure of 1000 ps. [36]. In the same way, other systems were built and operated in the program.

## 3. Materials and Methods

### 3.1. Materials

Natural rubber was obtained from Shanghai Acmec Biochemical Co., Ltd. (Shanghai, China). Syringic acid and erucic acid were supplied by Shanghai Aladdin Biochemical Technology Co., Ltd. (Shanghai, China) Silica was purchased from Guangzhou New Rare Metallurgical Chemical Co., Ltd. (Guangzhou, China). The other chemicals were commercially available in industrial grade.

### 3.2. Preparation of NR Composites

According to the ingredients in Table 1, to prepare the NR samples, the above components are placed on the open mill for specific mixing. The optimum cure time (*t*_90_) for NR samples was tested on an MDR2000 vulcanizer (ALPHA, Naperville, IL, USA) at 143 °C. Finally, the NR samples were hot-pressed and vulcanized on a flat vulcanizing machine at 143 °C for *t*_90_ + 2 min to gain NR composites.

### 3.3. Measurements and Characterization

#### 3.3.1. DPPH (2,2-Diphenyl-1-picrylhydrazyl) Radical Scavenging Test

The free radical scavenging activity of phenolic AOs is generally measured quantitatively by DPPH radical scavenging experiment. The DPPH radical has a single electron and a strong absorption peak at 517 nm. Therefore, the radical scavenging ability of AOs can be tested by measuring the change in absorbance of the DPPH solution at 517 nm [37]. First of all, we configure ethanol solutions of antioxidants and DPPH at a concentration of 5 × 10^−3^ mol/L. Afterward, 0.1 mL of antioxidant solution and DPPH solution are added sequentially to a quartz cuvette containing 3 mL ethanol. Finally, the quartz cuvette with the mixed solution is placed in the dark for 30 min, and then, the quartz cuvette with the mixed solution is put into the UV–vis spectrophotometer (UV-2600, Shimadzu, Japan) to measure its absorbance [38]. The test is repeated three times, and the final results are averaged. The DPPH radical scavenging rate is calculated as shown in Equation (2).

(2) $$\mathrm{DPPH}\;\mathrm{scavenging}\;\mathrm{activity}\left(\%\right)=\frac{A_{C}-A_{S}{}_{\;}}{A_{C}}\times 100\%$$

where *A_C_* represents the absorbance of the control and *A_S_* represents the absorbance of the sample.

#### 3.3.2. Accelerated Thermal-Oxidative Aging Experiments

The samples of NR composite are placed in an air circulation heated cabinet oven (Argentox, 3 MR-3RVB-140, Glinde, Germany) at a temperature of 100 °C for accelerated thermal-oxidative aging. The NR samples are taken out at different aging times (1, 3, and 5 days) for further characterization tests.

#### 3.3.3. Mechanical Property Test

Tensile measurement tests on dumbbell-type samples are performed using the universal testing machine (Zwick, Z005) with a velocity of 500 mm·min^−1^. All tests are performed at room temperature, and each datum is the average of five specimens.

#### 3.3.4. Fourier Transform Infrared Spectroscopy (FT-IR)

The functional group changes of NR composites during aging are detected by Attenuated total reflectance (ATR) mode using an FT-IR spectrometer (Bruker VERTEX 70, Germany). The wavenumber ranged from 4000 to 600 cm^−1^ with a resolution of 4 cm^−1^.

## 4. Results and Discussion

### 4.1. Hydrogen Dissociation Energy

**Figure 4:**
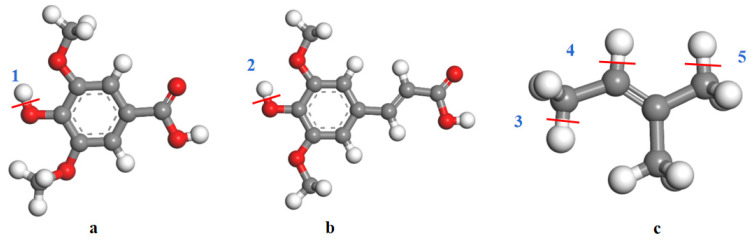
The bond-breaking positions of hydrogen in (**a**) syringic acid, (**b**) erucic acid, and (**c**) natural rubber repeat unit.

The bond-breaking positions of syringic acid, erucic acid, and natural rubber repeat units are shown in Figure 4. According to the results in Table 2. The *BDE* values of both syringic acid and erucic acid are less than those of NR, indicating that the O−H bond of syringic acid and erucic acid will preferentially dissociate. Therefore, antioxidants syringic acid and erucic acid can protect NR. Furthermore, the *BDE* value of syringic acid is higher than that of erucic acid. The *BDE* results indicate that erucic acid has higher activity in scavenging free radicals.

### 4.2. Dispersion and Migration of Antioxidants

In addition to the above-mentioned effect of dissociation energy on the antioxidant properties of antioxidants, the physical aspects of the antioxidant properties of antioxidants are equally important, such as the dispersibility and migration of antioxidants. Therefore, suitable dispersibility and migration resistance become a basic requirement for screening antioxidants. With the help of MD simulation, the compatibility of different substances can be predicted by calculating the solubility parameters of the substances. It is known from the semi-empirical approach description that Δ*δ* = |*δ*_antioxidant_ − *δ*_NR_| if the difference between the solubility parameters of AO and NR is less than 2.05 (J·cm^−3^)^1/2^, they are absolutely compatible, i.e., the closer the solubility parameter between AO and NR, the compatibility between AO and NR. *δ* is expressed as the square root of the cohesive energy density (*CED*).

(3) $$\delta =\sqrt{CED}=\sqrt{\frac{E_{coh}}{V}}=\sqrt{\frac{\Delta H_{vap}-RT}{V}}$$

where *E_coh_* means the cohesive energy, Δ*H_vap_* means the enthalpy of vaporization, *V* means the mole volume, *T* means the absolute temperature, and *R* means gas constant.

The simulation results of the solubility parameters of SA, EA, and NR are presented in Table 3. The simulated value of NR is 16.38 (J·cm^−3^)^1/2^, and the experimental value is 16.2–17.0 (J·cm^−3^)^1/2^, which are very close to each other [39], indicating that molecular simulation as a scientific research tool and its calculation results are credible. In Table 3, the solubility differential between selected antioxidants and NR is less than 2.05 (J·cm^−3^)^1/2^, and EA is smaller than SA, predicting that EA might achieve better dispersion in the NR matrix.

Furthermore, with the help of the MD simulation, dynamic processes of antioxidants in the rubber matrix can be visualized. The *MSD* curve can quantitatively respond to the migration of antioxidants in the NR matrix, which is calculated by the following equation.

(4) $$MSD=\frac{1}{N}\overset{N}{\underset{i=1}{\sum }}<|r_{i}\left(0\right)-{r_{i}\left(t\right)|}^{2}>$$

where *N* means the total number of atoms, *r_i_*(0) denotes the initial position of atom *i*, *r_i_*(*t*) denotes the position of atom i after time t, $|r_{i}\left(0\right)-r_{i}\left(t\right)|$ denotes the displacement of the atom during time *t,* and the bracket <> means the average square of the displacement for atom *i*.

**Figure 5:**
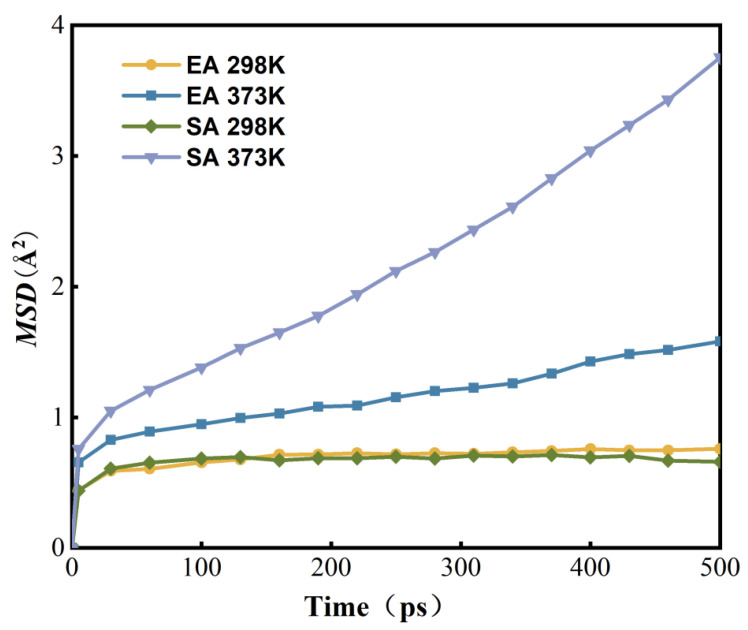
*MSD* curves of SA and EA in NR matrix.

Figure 5 shows the *MSD* curves of SA and EA in the NR matrix at different temperatures. The *MSD* value of EA and SA is not much different at 298 K, and the *MSD* value of EA is less than SA at 373 K. In addition, the *MSD* values at 373 K are higher than those at 298 K, indicating that the antioxidants migrate faster at high temperatures, agreeing with the fact that the antioxidant molecules migrate faster at the high temperature. Since the *MSD* value of EA is smaller, this indicates that EA has a higher resistance to migration and can exist more stably in the NR.

### 4.3. Oxygen Permeability

According to the RH aging reaction in Figure 1, we know that oxygen, a significant factor in material aging, can react with alkyl radicals (R·) to form very oxidizing peroxyl radicals (ROO). The production of peroxyl radicals leads to a range of following aging reactions. Thus, the barrier performance of the material to oxygen is a very important physical factor. Oxygen permeability is a key physical parameter of antioxidant-protected NR materials, and the formula is as follows:

*P* = *D* × *S* (5)

where *P*, *D*, and *S* represent the permeability coefficient, diffusion coefficient, and solution coefficient, respectively. The value of *D* is calculated based on the Einstein equation [40]:

(6) $$D=\frac{1}{6N}\underset{t\to \infty }{lim}\frac{d}{dt}\overset{N}{\underset{i=1}{\sum }}<|r_{i}\left(0\right)-{r_{i}\left(t\right)|}^{2}>$$

where *N* is the total number of selected oxygen molecules in the whole system. As for the calculation of the value of *S*, it is necessary to use the dual-mode sorption model.

(7) $$C=K_{DP}+\frac{C_{H}bp}{1+bp}$$

Then, *S* can be deduced from the following expression:

(8) $$S=\underset{P\to 0}{lim}(\frac{C}{P})=K_{D+C_{H}b}$$

Table 4 shows the calculation results of relevant parameters such as permeability coefficient (*P*). It can be observed that the permeability coefficients of the SA/NR system and the EA/NR system are lower than those of the NR system. It indicates that the addition of antioxidants improves the O_2_ barrier properties of the NR composites. Combining the previous results of the solubility parameters shown in Table 3 and the *MSD* curves shown in Figure 5, it can be predicted that syringic acid and erucic acid can be stably dispersed in the NR matrix with suitable migration resistance and oxygen barrier properties. In addition, the antioxidant effect of erucic acid was predicted to be better than that of syringic acid. Therefore, the following section will develop an experimental verification based on this prediction.

### 4.4. Analysis of Antioxidant Activity

**Scheme 1:**
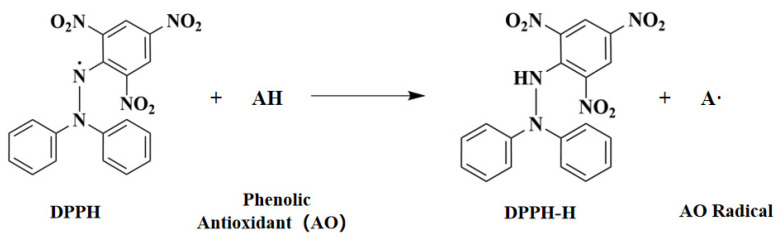
Principal mechanism of radical scavenging reaction.

The radical scavenging ability of antioxidants is usually measured by DPPH radical scavenging assay [22]. The main mechanisms of the DPPH radical scavenging reaction are as Scheme 1.

**Figure 6:**
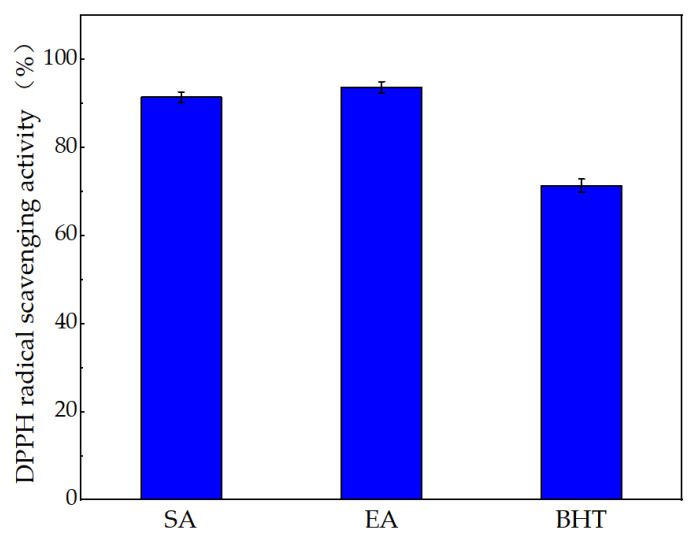
DPPH radical scavenging rates of syringic acid (SA), erucic acid (EA), and BHT.

In fact, the essence of the reaction is the dissociation of the H atom from the phenolic hydroxyl group (-OH) in the phenolic antioxidant to react with the DPPH radical, which is the same as the reaction of the peroxy radical in the RH. Therefore, DPPH radical scavenging assay can reflect the radical scavenging activity of antioxidants to a certain extent. It is worth mentioning that the synthetic antioxidant BHT is added as a reference control in this test. From the results shown in Figure 6, the antioxidant activities of both syringic acid and erucic acid are higher than those of the synthetic antioxidant BHT. Among them, the free radical scavenging rate of erucic acid is 93.7%, slightly higher than the free radical scavenging rate of syringic acid is 91.5%, verifying the predictions of molecular simulations.

### 4.5. Analysis of Mechanical Properties of NR Composites

**Figure 7:**
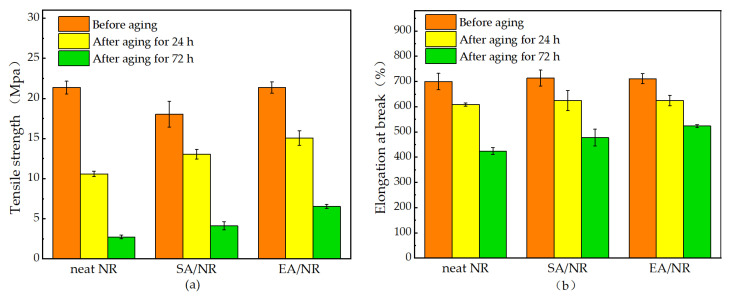
The mechanical properties of NR and AO/NR systems at different aging times: (**a**) tensile strength; (**b**) elongation at break.

The mechanical properties are important parameters to evaluate the rubber material in the process of use. It is necessary to test the mechanical properties of NR composites. Figure 7 shows the changes in the mechanical properties of NR and AO/NR systems. After 24 h of aging, the tensile strengths of SA/NR and EA/NR systems are 23.1% and 42.3% higher than that of the NR systems, indicating that the SA/NR and EA/NR systems have suitable anti-aging effects in the short-term. In addition, as far as elongation at break is concerned, the change in elongation at break of the AO/NR system is not much different from that of the NR system before and after aging, indicating that the effect of adding antioxidants on elongation at break is minimal. Therefore, the addition of antioxidants is an improvement in the mechanical properties of NR materials.

### 4.6. Microstructure Analysis

**Figure 8:**
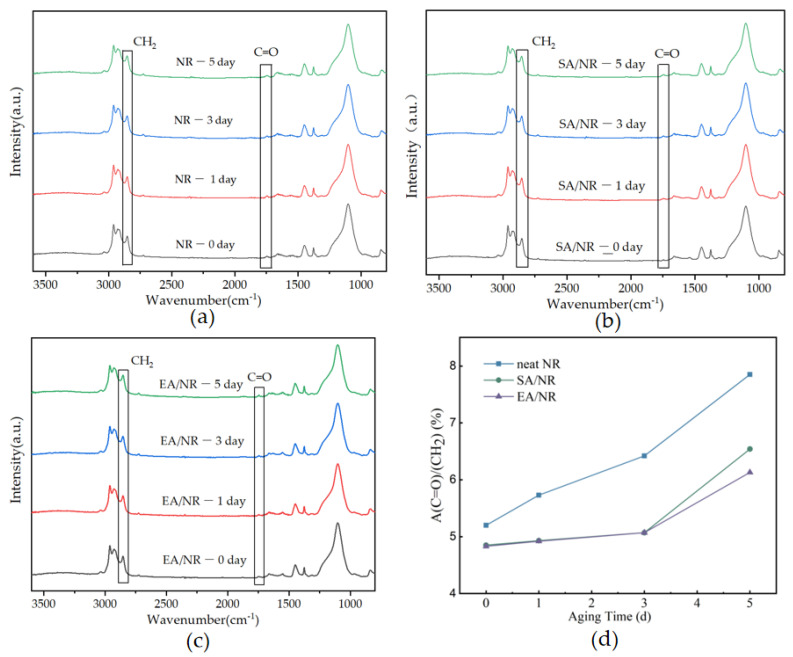
(**a**–**c**) The FT-IR spectra of NR composite before aging and after aging 1, 3, and 5 days and (**d**) the carbonyl increase ratio of A_(C=O)_/A_(CH2)_ for NR and AO/NR composites at 100 °C.

NR composites usually generate many oxidation products, such as ethers, carbonyl, and lactones, after thermal-oxidative aging. As illustrated in Figure 8a–c, the changes in carbonyl content of the NR system and different AO/NR systems at different aging times (1, 3, 5 days) were observed under the FT-IR spectra. It was demonstrated that a carbonyl (C=O) absorption peak at approximately 1745 cm^−1^, and CH_2_ has a stretching vibration peak at 2848 cm^−1^ and that this peak does not change with the aging of the material [41]. Therefore, the ratio of A_(C=O)_/A_(CH2)_ can reveal the degree of thermal-oxidative aging of NR materials. It can be seen in Figure 8d, the NR system, that the carbonyl content increases significantly with the increase in aging time. On the contrary, the rate of increase in carbonyl content in the AO/NR system decreased significantly. From the experimental results, antioxidants can delay the increase in the carbonyl ratio. Moreover, SA and EA showed comparable anti-aging effects at 1 and 3 days of aging, and EA showed better anti-aging effects as the aging time increased.

## 5. Conclusions

In this study, we studied the influence of syringic acid and erucic acid on the antioxidant properties of NR by combining experimental and computational methods. The antioxidant capacity was simulated and predicted. First, the values of *BDE* were obtained by QM simulations, and the mechanism of antioxidant protection against NR was explained. Then, the effects of syringic acid and erucic acid on NR composites were analyzed using MD simulations to explore the compatibility differences, the migration resistance of AO, and the barrier properties of O_2_ in the NR matrix. The theoretical results predicted that both syringic acid and erucic acid can be stably dispersed in the NR matrix and have high antioxidant activity and O_2_ barrier properties. As for the aspect of the experiment, radical scavenging experiments verified the antioxidant activity of the antioxidants. SA and EA possess higher antioxidant activity compared to the synthetic antioxidant BHT. Mechanical properties tests showed that the SA/NR and EA/NR composites increased the tensile strength by 23.1% and 42.3%, respectively, compared to the pure NR composite after one day of aging. Microstructure analysis of NR composites showed that the addition of SA and EA can effectively delay the increase in oxidation products in the composite.

Given these results, syringic acid and erucic acid shows suitable thermal oxidation stability during the rubber aging process, which means that syringic acid and erucic acid should have a broader application prospect. Moreover, molecular simulations provide a theoretical basis for the quantitative study of the anti-aging mechanism of antioxidants in natural rubber materials and provide guidelines for screening suitable antioxidants for other systems.

## Figures and Tables

**Table 1:** The formulas of AO/NR composites.

Ingredient (phr) ^1^	Samples		
	NR	SA/NR	EA/NR
NR	100	100	100
SA	0	1.2	0
EA	0	0	1.2

^1^ Other rubber additives: silica, 30 phr (parts per hundreds of rubber); ZnO, 5 phr; stearic acid, 2 phr; S, 2 phr; CZ, 1.5 phr.

**Table 2:** The hydrogen dissociation energies for SA, EA, and NR.

Dissociation Position	1	2	3	4	5
Δ*G*^298K^ (kJ·mol^−1^)	98.02	78.97	375.05	411.06	342.20

**Table 3:** Solubility parameters for SA, EA, and NR.

Samples	Solubility Parameter *δ* (J·cm^−3^)^1/2^		
	Simulation	Experiment	Δ*δ* = |*δ*_antioxidant_ − *δ*_NR_|
NR	16.38	16.2–17.0	-
SA	14.77	-	1.61
EA	15.42	-	0.96

**Table 4:** The *D*, *S*, and *P* values for O_2_ in NR and AO/NR systems at 298K.

System	*D* (10^−6^ cm^2^ s^−1^)	*S* (10^−3^ cm^3^ (STP) cm^−3^ kPa^−1^)	*P* (10^−9^ cm^2^ s^−1^ kPa^−1^)
NR	3.7	1.70	6.29
SA/NR	1.8	2.99	5.38
EA/NR	1.9	0.67	1.27

## Data Availability

Not applicable.
